# Investigation of chemoresistance to first-line chemotherapy and its possible association with autophagy in high-risk neuroblastoma

**DOI:** 10.3389/fonc.2022.1019106

**Published:** 2022-10-20

**Authors:** Tingting Chen, Chenggong Zeng, Zhuoran Li, Juan Wang, Feifei Sun, Junting Huang, Suying Lu, Jia Zhu, Yizhuo Zhang, Xiaofei Sun, Zijun Zhen

**Affiliations:** ^1^ Department of Pediatric Oncology, Sun Yat-sen University Cancer Center (SYSUCC), Guangzhou, China; ^2^ State Key Laboratory of Oncology in South China, Sun Yat-sen University Cancer Center (SYSUCC), Guangzhou, China; ^3^ Collaborative Innovation Center for Cancer Medicine, Sun Yat-Sen University Cancer Center, Guangzhou, China

**Keywords:** Autophagy, chemoresistance, neuroblastoma, induction chemotherapy, chloroquine

## Abstract

High-risk neuroblastoma (NB) is sensitive to chemotherapy but susceptible to chemoresistance. In this study, we aimed to analyze the incidence of chemoresistance in high-risk NB patients and to explore the role of autophagy in NB chemoresistance. We retrospectively analyzed the incidence of changing the chemotherapy regimen due to disease stabilization or disease progression during induction chemotherapy in high-risk NB patients, which was expressed as the chemoresistance rate. The autophagy levels were probed in tumor cells exposed to first-line chemotherapy agents. The sensitivity of tumor cells to chemotherapy agents and apoptosis rate were observed after inhibiting autophagy by transfection of shRNA or chloroquine (CQ). This study included 247 patients with high-risk NB. The chemoresistance rates of patients treated with cyclophosphamide + adriamycin + vincristine (CAV) alternating with etoposide + cisplatin (EP) (Group 1) and CAV alternating with etoposide + ifosfamide + cisplatin (VIP) (Group 2) was 61.5% and 39.9% (P = 0.0009), respectively. Group 2 had better survival rates than group 1. After exposure to cisplatin, cyclophosphamide, and etoposide, the autophagy-related proteins LC3-I, LC3-II, and Beclin-1 were upregulated, and the incidence of autophagy vesicle formation and the expression of P62 were increased. Chemotherapeutic agents combined with CQ significantly increased the chemotherapeutic sensitivity of tumor cells and increased the cell apoptosis. The downregulated expression of Beclin-1 increased the sensitivity of tumor cells to chemotherapeutics. Our results suggest that increasing the chemotherapy intensity can overcome resistance to NB. Inhibition of autophagy is beneficial to increase the sensitivity of NB to chemotherapy agents.

## Introduction

Neuroblastoma (NB) originates from primitive neural crest cells. It is the most common pediatric extracranial solid tumor with highly aggressive, diverse biological behaviors and rapid progression. Approximately 50% of patients were accompanied by distant metastasis at diagnosis (1). Under multimodality treatments, the 5-year survival rate of non-high-risk patients exceeded 90% (2, 3). However, the prognosis of high-risk patients remains poor, seriously threatening children’s lives (2).

The treatment of high-risk NB patients includes induction chemotherapy, local control, consolidation, and maintenance therapy, among which induction chemotherapy plays an important role. Induction chemotherapy can reduce the tumor burden, clear metastasis, improve the surgical resection rate, and evaluate the tumor sensitivity to agents. In addition, the efficacy of induction chemotherapy can also predict the long-term survival (4). Neuroblastomas are remarkably heterogeneous, with distinct yet variable biological and clinical characteristics, including localized or widely disseminated disease that spontaneously resolves, low-to-intermediate-risk tumours that can be surgically resected with or without the need for postoperative chemotherapy, and high-risk disease, which is often lethal, so some patients is sensitive to chemotherapy,but some patients show chemoresistance at the beginning or during treatment (5). Approximately 71%–85% of high-risk patients can achieve partial or complete response after induction therapy, but 20% of high-risk NB patients have little response to standard chemotherapy (6).

Chemoresistance has become one of the main obstacles to NB treatment. The mechanism of chemoresistance in NB is complex, and multiple mechanisms may coexist (7–10). In recent years, studies have found that autophagy is a core molecular pathway for maintaining cellular and biological homeostasis, participating in tumor recurrence and therapeutic resistance events, and plays a key role in promoting cancer growth (7). Many preclinical studies have confirmed that the chemoresistance of tumor cells to chemotherapy agents such as adriamycin, cisplatin, temozolomide, paclitaxel, and cytarabine is related to autophagy; hence, tumor chemoresistance can be reversed by regulating autophagy (8–12).

First-line chemotherapy regimen of high-risk NB often contains five–six chemotherapy agents (13, 14). Data on autophagy induced by use of each chemotherapy agent and its effect on chemoresistance in NB during treatment are limited. Sun Yat-sen University of Cancer Center used modified CAV/EP regimen for the treatment of high-risk NB patients. In 2013, ifosfamide was introduced and evolved into CAV/VIP regimen. This study aimed to examine the incidence of chemoresistance at different time points during the course of high-risk NB chemotherapy; explore the relationship between use of first-line chemotherapy agents, autophagy, and chemoresistance; and address the issues related to autophagy and chemoresistance.

## Materials and methods

### Patients

Patients with high-risk NB and who were younger than 18 years old at initial diagnosis from January 2006 to February 2019 at Sun Yat-sen University of Cancer Center were enrolled in this study. NB was confirmed by pathological examination. All children did not show significant organ function impairment caused by other serious diseases before treatment. This study involving human participants was reviewed and approved by the ethics Committee of Sun Yat-sen University of Cancer Center (approval no. B2022-291-01), and was conducted according to the principles of the Declaration of Helsinki. All children’s guardians signed an informed consent.

### Staging and risk stratification

Cancer staging was determined based on the International Neuroblastoma Staging System (INSS) (15). Improvements were made to the Children’s Oncology Group (COG) risk stratification method (16) used in this study. The following individuals were classified as high-risk patients: those with (1) INSS stage 4 NB who were aged 1–18 years with and without MYCN amplification; (2) INSS stage 2, 3, 4, or 4S NB with MYCN amplification; and (3) INSS stage 3 who were aged 1–18 years with and without MYCN amplification and unfavorable International Neuroblastoma Pathology Classification.

### Treatment

All patients received induction chemotherapy after diagnosis. The patients were divided into two groups according to their induction chemotherapy regimen. Before 2013, patients were treated with alternating regimen of cyclophosphamide + adriamycin + vincristine (CAV) and etoposide + cisplatin (EP) (Group 1). After 2013, patients were treated with alternating regimen of CAV and etoposide + ifosfamide + cisplatin (VIP) (Group 2). The specific chemotherapy regimen is shown in **Table 1**. Both regimens were administered every 3 weeks, and the efficacy was evaluated after two courses of treatment using the WHO efficacy evaluation method (17). The incidence of changing chemotherapy regimen due to stable disease (SD) or progressive disease (PD) after 2, 4 and 6 courses of induction therapy was calculated in each group, which was defined as the chemoresistance rate in this study.

**Table 1:** Chemotherapy regimens for high-risk neuroblastoma.

Regimens	Dosage and usage
CAV	Cyclophosphamide (CTX)1000 mg/m^2^,iv drip, D1
	Adriamycin (ADR) 50 mg/m^2^,iv, D1
	Vincristine (VCR) 1.5mg/m^2^(≤2mg)iv, D1
EP	Etoposide (VP16)100 mg/m^2^,iv drip, D1-5
	Cisplatin (DDP) 20(mg/m^2^), iv drip, D1-5
CAV^a^	Cyclophosphamide (CTX)1000mg/m^2^, iv drip, D1-2
	Adriamycin (ADR) 50 mg/m^2^,iv, D1
	Vincristine (VCR) 1.5 g/m^2^(≤2mg)iv, D1
VIP	Etoposide (VP16)100 mg/m^2^,iv drip, D1-4
	Ifosfamide (IFO)150 mg/m^2^,iv drip, D1-4
	Cisplatin (DDP) 25 mg/m^2^ iv drip, D1-4

a: dose of CTX was escalated.

After 4 to 6 courses of induction chemotherapy, local treatment was adopted according to its efficacy. The primary lesion was resected if appropriate. Radiotherapy was administered in patients with postoperative tumor residue, using a dose of 25–35 Gy. Some patients underwent consolidation therapy with high-dose chemotherapy along with transplantation of autologous hematopoietic stem cells. Before 2013, oral retinoic acid was used for maintenance therapy at a dose of 160 mg/m^2^/d, followed by 7 days of oral retinoic acid and 7 days of treatment cessation for a year. Since 2013, maintenance treatment has been switched to traditional chemotherapeutic treatment with long-term metronomic therapy, administered orally in small doses, for 1 year. See previous study for details (18).

The salvage chemotherapy regimens used for refractory or recurrent patients include IFO + carboplatin (IE regimen), VCR + irinotecan + temozolomide (VIT regimen), and CTX + topotecan (CT regimen).

### Cells culture

Human SH-SY5Y cells (Nanjing Cobb Biotechnology Co, LTD, Nanjing, China) were cultured in MEM-F12 medium supplemented with 10% fetal bovine serum, 1% non-essential amino acid, 1% sodium valproate, 100 units/mL of penicillin, and 100µg/mL of streptomycin (Inweigenic Trading Co, Ltd., Shanghai, China). These cells were cultured in a 5% CO_2_ incubator at 37°C, and the solution was changed every other day. Cells in the logarithmic growth period was used for the experiment.

### Cell counting kit 8

The sensitivity of cells to agents was determined using the Cell Counting Kit 8 (CCK-8) reagent (APExBIO, Guangzhou, China). SH-SY5Y cells were digested, centrifuged, and seeded in 96-well plates containing 200 μl of culture medium and 10,000 cells per well. After incubating the culture plate overnight, different concentrations of cyclophosphamide, cisplatin, and etoposide were added. Forty-eight hours after exposure to these agents, the original culture medium was discarded, and 100 μl of 10% CCK8 solution was added to each well. After 3–4 hours of incubation, the absorbance was measured at a wavelength of 450 nm in a microplate reader.

### Western blot

The cells were collected in each group, rinsed with cold Phosphate-Buffered Saline (PBS) twice, and then added with protein lysate to extract the protein. The protein purity was determined using the Bradford method. After protein denaturation, 20 μg of protein was isolated by SDS-PAGE; transferred to the PDVF membrane, which was sealed with defatted milk powder for 2 h, added with primary antibody with appropriate dilution concentration, and incubated overnight in a 4° shaking bed; and then rinsed with TBS-T four times for 10 min each time. Then, the second antibody was added at an appropriate diluted concentration; incubated for 2 h; rinsed with TBS-T four times, 10 minutes each time; stained; and finally exposed.

### Plasmid transfection

The cells were seeded in a six-well plate at 5×10^5/well. After cell adherence, GFP-RFP-LC3 plasmid was transferred to cells according to the lentivirus plasmid transfection protocol, and cell lines expressing GFP-RFP-LC3 was established. The transfected tumor cells were treated with DDP, CTX, VP16, IFO, ADR and VCR, and the autophagy flow was observed under confocal microscope after 48 h. The transfection of Beclin-1 shRNA plasmid was performed the same as above; the cell line with low expression of Beclin-1 was established; the transfected tumor cells were treated with DDP, CTX, and VP16; and the half maximal inhibitory concentration (IC50) of each agent was determined.

### Confocal microscopy detection

SH-SY5Y cells transfected with GFP-RFP-LC3 plasmid were digested, centrifuged, and seeded in a 15-mm confocal dish containing 1.5 ml of culture medium and 30,000 cells per dish. After the dishes were incubated in an incubator overnight, cyclophosphamide, cisplatin, etoposide, ifosfamide, doxorubicin, and vincristine were added individually and observed under a confocal zeise LSM880 microscope for 48 h. First, the field of vision was adjusted under low power lens, and then the 60X oil lens was used for observation.

### Flow cytometry

The cells were exposed to cyclophosphamide, cisplatin, etoposide, and their combinations with CQ. After 48 h of exposure, the supernatant was discarded, and the cells were collected and stained with FITC/PI apoptotic reagent. The apoptotic cell ratio of each group was observed using an SP6800 spectral flow cytometer.

### Statistical analysis

All data were analyzed using SPSS 25.0 for Windows (SPSS Inc.). The overall survival (OS) and event-free survival (EFS) were analyzed using the Kaplan-Meier method and compared using log-rank test. OS was defined as the time from the diagnosis to death or final follow-up. Event-free survival was defined as the time from diagnosis to tumor recurrence, progression, second tumor, death from any cause, or final follow-up. The data were presented as mean ± SD. The means of the samples were compared using an unpaired, two-tailed Student’s t test, or one-way analysis of variance. A threshold P value of <.05 indicated statistical significance.

## Results

### Patients’ characteristics

A total of 247 children with high-risk NB were enrolled in this study, with a median age of 3.8 years (0.7–15.0 years). Of them, 160 were male patients and 87 were female patients. Among the 247 patients, 109 were included in Group 1 and 138 in Group 2. The specific clinical features are shown in **Table 2**.

**Table 2:** Clinical characteristics of high-risk neuroblastoma children.

Characteristics	Group 1	Group 2	*P* value
	n (%)	n (%)	
Age (year)			
<1.5	9 (8.3)	14 (10.1)	0.664
1.5-18	100 (91.7)	124 (89.9)	0.664
Sex			
male	66 (60.6)	94 (68.1)	0.229
female	43 (39.4)	44 (31.9)	0.229
Primary site			
Abdomen	102 (93.6)	124 (89.9)	0.362
Chest	6 (5.5)	9 (6.52)	0.795
Pelvic	1 (0.9)	3 (2.2)	0.632
Neck	0 (0)	1 (0.7)	1
Other	0 (0)	1 (0.7)	1
Pathology			
Neuroblastoma	89 (81.6)	107 (77.5)	1
Ganglion neuroblastoma	20 (18.4)	31(22.5)	0.527
Stage			
III	10 (9.2)	8 (5.8)	0.334
IV	99 (90.8)	130 (94.2)	0.334
MYCN			
+	26 (23.9)	26 (18.8)	0.350
–	36 (33.0)	70 (50.7)	0.006
Unmeasured	47 (43.0)	42 (30.4)	0.045

Group 1: 109 patients were treated with CAV/EP induction chemotherapy; Group 2: 138 patients were treated with CAV/VIP induction chemotherapy.

### Chemotherapy response rate

At the end of induction chemotherapy, in Group 1, 3 patients (2.8%) achieved complete response (CR), 77 patients (70.6%) achieved partial response(PR), 28 patients (25.7%) had SD, and 1 patient (0.9%) had PD; the objective response rate (CR + PR) was 73.5%. In Group 2, 16 patients (11.6%) achieved CR, 105 patients (76.1%) achieved PR, 16 patients (11.6%) had SD, and 1 patient (0.7%) had PD; the objective response rate was 87.7%.

### Correlation between chemotherapy intensity and chemoresistance rate

We retrospectively analyzed the chemoresistance rate in 247 high-risk NB patients at different time points of induction chemotherapy, and found that the overall chemoresistance rates of Group 1 and Group 2 were 61.5% and 39, 9%, respectively (P = 0.0009). The chemoresistance rate of Group 1 was higher than that of Group 2 after 4 courses (20.2% vs. 8.0%, P = 0.007) after comparing the chemoresistance rate of the two groups at each time point. No significant difference was observed in the chemoresistance rates at other time points (**Table 3**).

**Table 3:** Comparison of chemoresistance rates among different chemotherapy regimens.

		Group 1	Group 2	*P* value
		n (%)	n (%)	
Non-resistance		*42 (38.5)*	83 (60.1)	0.0009
Resistance	After 2 courses	8 (7.3)	3 (2.2)	0.064
	After 4 courses	22 (20.2)	11 (8.0)	0.007
	After 6 courses	37 (37.4)	41 (29.7)	0.262
	Accumulation	67 (61.5)	55 (39.9)	0.0009
Total		109 (100)	138 (100)	–

Group 1: CAV/EP for induction chemotherapy; Group 2: CAV/VIP was used for induction chemotherapy.

### Benefit of increasing the chemotherapy intensity in patients’ survival

**Figure 1:**
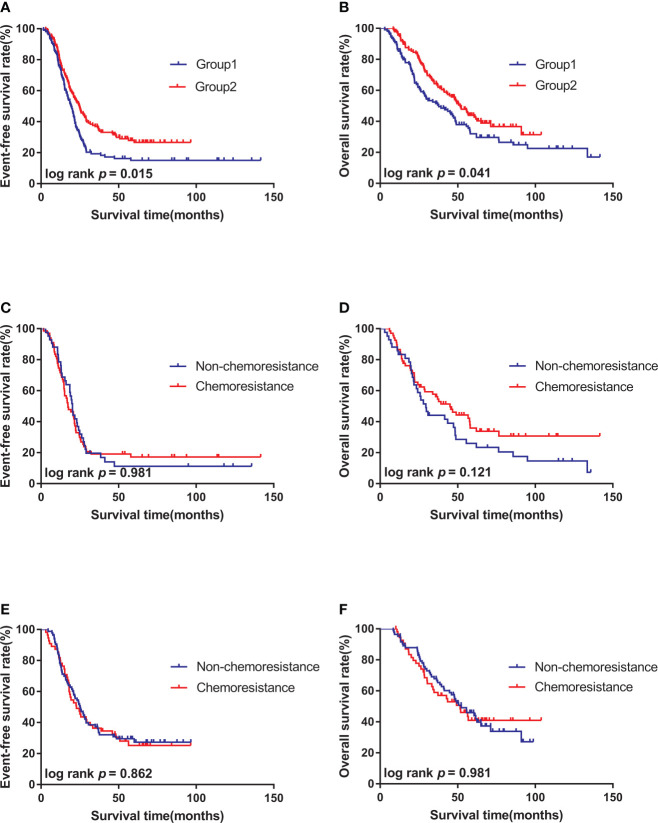
Survival curves of high-risk neuroblastoma of different subgroups. **(A)**. The 3-year EFS of patients in Group 2 (n = 138) was better than that in Group 1(n = 109). **(B)**. The 3-year OS of patients in Group 2 (n = 138) was better than that in Group 1(n = 109). **(C)**. There was no significant difference in 3-year EFS between chemoresistance (n = 67) and non-chemoresistance (n = 42) patients in Group 1. **(D)**. There was no significant difference in 3-year OS between chemoresistance (n = 67) and non-chemoresistance (n = 42) patients in Group 1; **(E)**. There was no significant difference in 3-year EFS between chemoresistance (n = 55) and non-chemoresistance (n = 83) patients in Group 2. **(F)**. There was no significant difference in 3-year OS between chemoresistance (n = 55) and non-chemoresistance (n = 83) patients in Group 2.

K-M survival analysis was performed to compare the effects of regimens with different intensities on patient’s survival. The median follow-up times were 55.5 months (3.0–141.8 months) for Group 1 and 49.4 months (8.9–103.8 months) for Group 2. The 3-year EFS rates were 36.9% in Group 2 and 19.3% in Group 1 (P = 0.015) (**Figure 1A**). The 3-year OS rates were 64.3% in Group 2 and 51.5% in Group 1 (P = 0.025) (**Figure 1B**).

### Effect of chemoresistance on patient’s survival

K-M survival analysis was performed to compare the effect of chemoresistance on the survival of patients treated with the same regimen. Results showed that the 3-year EFS (23.0% vs. 19.1% P = 0.981) and 3-year OS (43.2% vs. 51.5% P = 0.121) of non-chemoresistance and chemoresistance patients in Group 1 had no statistical significance (**Figures 1C, D**). No statistical significance was also observed in the 3-year EFS (37.3% vs. 36.4% P = 0.862) and 3-year OS (58.9% vs. 67.8% P = 0.981) of non-chemoresistance and chemoresistance patients in Group 2 (**Figures 1E, F**).

### Adverse reactions

Two groups showed good tolerance to chemotherapy, with myelosuppression being the most common adverse reaction. The incidence rates of grade III–IV myelosuppression were 69.7% in Group 1 and 83.3% in Group 2. No significant difference was found between the two groups in terms of the incidence of cardiotoxicity, impaired liver function, impaired kidney function, nausea, vomiting, abdominal pain, and other gastrointestinal symptoms. No treatment-related deaths occurred in either group.

### Effect of cisplatin, etoposide, and cyclophosphamide in the expression of autophagy-related proteins in NB cells

**Figure 2:**
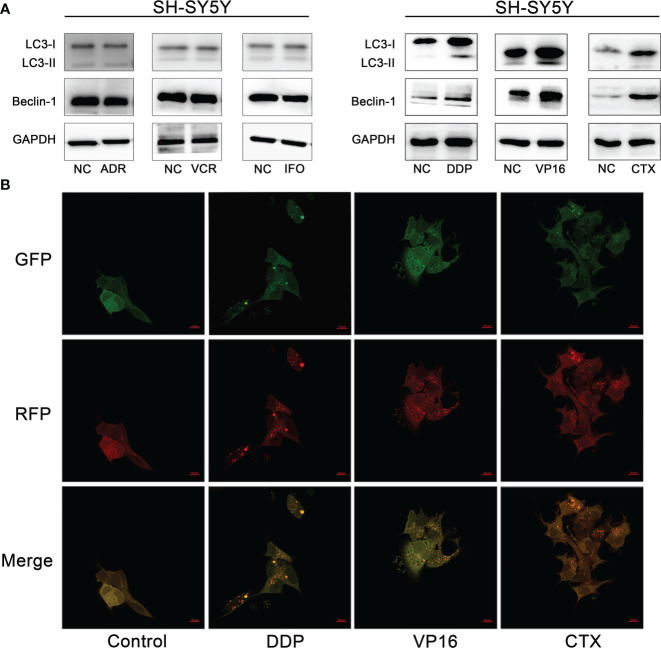
Autophagy induced by chemotherapy agents in neuroblastoma cells. **(A)** Western blot analysis showed that DDP, VP16 and CTX induced increased expression of LC3-I, LC3-II and Beclin-1 after SH-SY5Y cells were treated with 6 chemotherapy agents. However, IFO, VCR and ADR showed no increase in autophagy related protein expression. **(B)** Confocal microscopy observed that autophagic vesicles increased significantly after tumor cells were exposed to chemotherapy agents (DDP, CTX, VP16) compared with the untreated group.

A preliminary experiment was carried out to determine whether the expression of autophagy-associated proteins can be induced after treatment with the six first-line chemotherapy agents in high-risk NB. The SH-SY5Y cells were treated with 1 μM of DDP, 0.5 μm of VP16, 1 mm of CTX, 1 mm of IFO, 25 nm of ADR, and 10 nm of VCR for 1 month; then, the proteins were extracted for Western blot analysis. The results showed that the protein expression levels of LC3-I, LC3-II, and Beclin-1 were upregulated after treatment with DDP, VP16, and CTX compared with the control group, while the protein expression levels of LC3-I, LC3-II, and Beclin-1 were not significantly changed after treatment with ADR, IFO, and VCR (**Figure 2A**). This finding suggests that 3 out of 6 first-line chemotherapy agents can induce autophagy in NB cells.

### Effect of chemotherapy in the formation of autophagic vesicles and autophagic flux in neuroblastoma cells

To further verify whether DDP, VP16, and CTX could induce autophagy in NB cells, we observed the fluorescence intensity changes of SH-SY5Y cells 48 hours after exposure to DDP (2 μm), VP16 (1 μm) and CTX (2 m) under a confocal microscope (**Figure 2B**). The results showed that the punctured structure of LC3 was significantly increased in the experimental group compared with that in the negative control (NC) cells, which is an important marker of autophagy induction. LC3 spots were used to evaluate the degree of autophagosome and autophagolysosome formation. Compared with the control group, the number of RFP+GFP+ yellow spots in the experimental group was significantly higher than that in the control group, indicating an increased autophagosome formation. The number of red spots in RFP+ GFP-2 in the experimental group was significantly higher than that in the control group, indicating an increased autophagic lysosome formation and autophagic flux. These results suggest that chemotherapeutic agents can induce an increase in the number of autophagic vesicles in NB cells.

**Figure 3:**
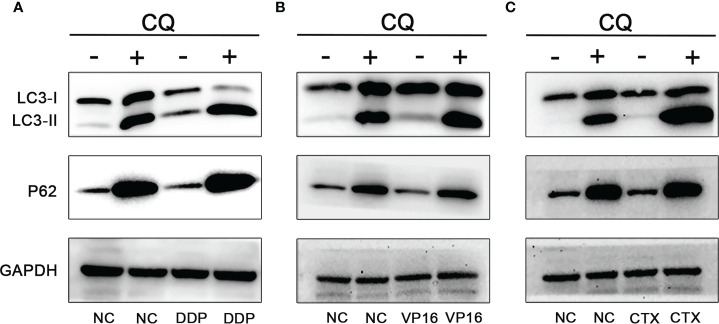
Effect of CQ on chemotherapeutic induced autophagy. Effect of CQ on autophagy after DDP **(A)**, VP16 **(B)** and CTX **(C)** acted on neuroblastoma, the expression of LC3-I and LC3-II increased, while the expression of P62 decreased. After combined with CQ, the expression of LC3-I and LC3-II further increased, and the latter increased more significantly. P62 expression level was increased due to inhibition of autophagy and decreased degradation.

To further determine the increase of autophagy flux in NB cells, the SH-SY5Y cells were treated with CQ (15 μm) combined with DDP (2 μm), VP16 (1 μm), and CTX (2 mm), and the proteins were extracted 48 hours after treatment. Western blot assay was used to detect the expression of LC3 and P62. Results showed that in the absence of CQ treatment, the expression levels of LC3-I and LC3-II in the NB cells exposed to chemotherapy agents were higher than those in the NC group, while the expression level of P62 was slightly lower than that of the NC group. After CQ treatment, the expression levels of LC3-I and LC3-II in NB cells exposed to chemotherapy agents were increased further, and the expression level of P62 was significantly higher than that of the unexposed group due to autophagy inhibition; this finding suggests that DDP, VP16, and CTX can induce the increase the number of autophagy vesicles in NB SH-SY5Y cells (**Figure 3**).

### Effect of autophagy inhibition on chemotherapeutic sensitivity and apoptosis

**Figure 4:**
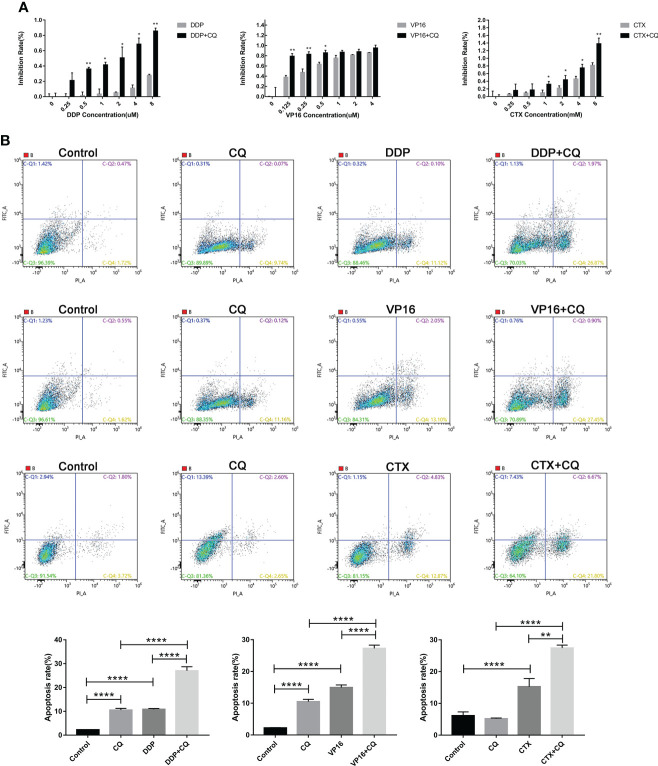
Inhibition of autophagy by CQ increases chemotherapy sensitivity and apoptosis. **(A)** CCK8 results showed that inhibition rate of SH-SY5Y cells exposed to CQ combined with chemotherapy agents was significantly higher than that of the group exposed only to chemotherapy agents, *P < 0.05, **P < 0.01. **(B)** Flow cytometry results showed that the apoptosis rate of SH-SY5Y cells exposed to CQ combined with chemotherapy agents, was significantly higher than that only exposed to chemotherapy agents. The data represent the mean ± SEM of three independent experiments. *P < 0.05, **P < 0.01, ****P < 0.0001, two-tailed t test.

The CCK8 method was used to detect the changes in cell viability in the group exposed to monotherapy and the group exposed to a combination of chemotherapy agents and CQ. Results showed that, using the same concentration of chemotherapy agents, the cell inhibitory effect of the combination of CQ and chemotherapy agents was significantly greater than that of the monotherapy group (**Figure 4A**). In order to further verify the sensitization effect of CQ on the chemotherapeutic agents, apoptosis experiments were conducted, and the results showed that the apoptosis rate of NB cells exposed to the combination group was significantly higher than that of the monotherapy group. The above results indicate that chemotherapy combined with CQ can increase the chemotherapy sensitivity and apoptosis of NB (**Figure 4B**).

### Effect of downregulation of Beclin-1 on chemoresistance

**Figure 5:**
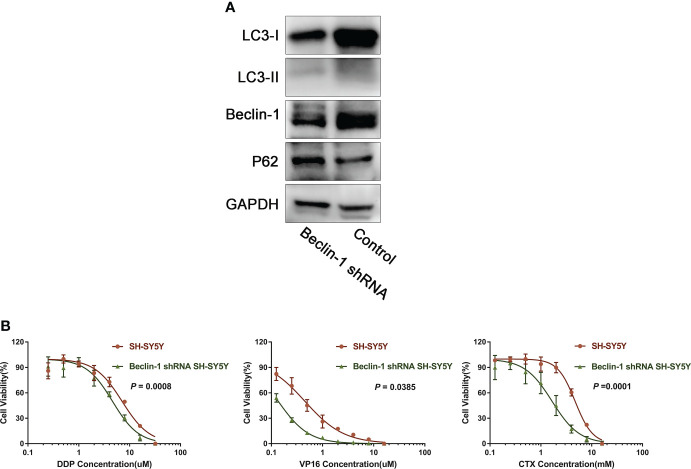
Down-regulation of Beclin-1 reverses chemoresistance of NB. **(A)** After transfection of Beclin-1 shRNA into SH-SY5Y cells, the expression of LC3-I and LC3-II decreased, while the expression of P62 increased. **(B)** After down-regulation of beclin-1 expression, IC50 of DDP, VP16 and CTX cells decreased compared with normal SH-SY5Y cells, The data represent the mean ± SEM of three independent experiments. P < 0.05, two-tailed t test.

In order to further explore the relationship between autophagy and chemoresistance, Beclin-1 shRNA was transfected into SH-SY5Y cells following the lentivirus transfection scheme. Results showed that the expression of autophagy-related proteins LC3-I and LC3-II decreased to varying degrees, while that of P62 increased (**Figure 5A**). Then, the IC50 values of DDP, VP16, and CTX in normal cells and Beclin-1 shRNA cells were measured. Results showed that the IC50 values of DDP, VP16, and CTX in NB cells significantly decreased after downregulation of Beclin-1 compared with that in NB cells without Beclin-1 knockdown (**Figure 5B**). This result showed that silencing the expression of Beclin-1 could enhance the sensitivity of cells to chemotherapy.

## Discussion

The chemoresistance to different intensities of induction chemotherapy for high-risk NB was analyzed in this study, and the different measures were preliminarily discussed. Compared with Group 1, the efficiency of high-intensity chemotherapy in Group 2 increased to 87.7%, indicating that NB was highly sensitive to chemotherapy, and the response rate was correlated with chemotherapy intensity. After high-risk NB is diagnosed, treatment with an effective chemotherapy can relieve the symptoms quickly, including emergency measures for some life-threatening cases (19). At the same time, specific indications are established for subsequent local treatment.

Although NB is highly sensitive to chemotherapy, many patients exhibited slower rate of tumor shrinkage as the chemotherapy progressed. In this study, 39.9%–61.5% of patients required to change their chemotherapy regimen before local treatment. Many studies have shown that the prognosis of NB patients with chemoresistance is poor (20, 21). Our study found no difference in survival between chemoresistance and non-chemoresistance patients in both groups, which may be attribute to the small sample size. Moreover, study bias may exist due to the limitations of a retrospective analysis, long research period, and inconsistent use of maintenance treatment among patients.

Nevertheless, chemoresistance has other adverse effects on NB therapy. Chemoresistance may delay the local treatment or affect the surgical resection rate, which are associated with poor prognosis (22). Due to chemoresistance, it is necessary to change other second-line chemotherapy, and its chemotherapy efficiency may not be superior to that of first-line chemotherapy. Frequent changes in chemotherapy regimen also increases the cumulative toxicity of chemotherapy. Replacement of regimens, delaying local treatment, and prolonging the treatment cycle also increase the economic and psychological burden of patients and increase the treatment cost.

Several previous studies focused on overcoming NB resistance to improve the antitumor efficacy (9, 21, 23, 24). Increasing the dose of chemotherapy but ensuring that the dose is still within the safe range to reduce the occurrence of chemoresistance is one of the primary solutions (25). Alkylation agents are one of the most important agents in the treatment of NB (26). In this study, the dose of cyclophosphamide was increased to 2 g/m^2^, and another alkylating agent ifosfamide was added to form a more intensive chemotherapy, which significantly reduced the chemoresistance rate. Although Group 2 had a higher incidence of myelosuppression and infection, the toxicity can be tolerated. Among the remaining treatments, using of long-acting G-CSF and performing peripheral blood tests more frequently are good options in reducing the incidence of myelosuppression and infections (27). A similar study was conducted by the International Society for Childhood Oncology in Europe, which reported a significantly improved objective response rate after using the high-intensity Rapid COJEC regimen (13).

Despite the increased intensity of chemotherapy, chemoresistance still occurred in more than one-third of patients during treatment; hence, development of other effective strategies to avoid resistance is still needed. We examined the level of autophagy induced by the components of high-risk NB first-line chemotherapy regimen; results showed that DDP, VP16, and CTX could increase the expression of autophagic protein, autophagic flow, and autophagosome in NB cells, while the other three agents did not significantly increase the level of autophagy after acting on the NB cells.

Since autophagy has been confirmed to be closely related to NB chemoresistance (28–31), further study was conducted to investigate the correlation of autophagy and chemoresistance to the three selected chemotherapy agents. Autophagy inhibitor chloroquine was used to block the effect of autophagosomes and lysosomes, resulting in the inhibition of the advanced stage of autophagy. After treating the cells with the three chemotherapy agents combined with CQ, not only did the cell viability decreased significantly, but the proportion of apoptosis also increased significantly. The sensitization of CQ on chemotherapy by inhibiting autophagy has been confirmed in many other tumor types (32–35). Our previous studies also found that paclitaxel, which is not commonly used in the treatment of NB, can induce autophagy-related chemoresistance in NB cells, and inhibition of autophagy can increase the tumor suppressive effect of paclitaxel, providing more agent options for NB treatment (11).

We further explored the regulatory mechanism of autophagy. After the expression level of Beclin-1 in NB cells was downregulated by shRNA, the expression levels of LC3-I and LC3-II decreased, the expression level of P62 was upregulated, and the sensitivity to chemotherapy increased, suggesting that Beclin-1 is an important regulatory gene for autophagy-related chemoresistance. The expression of Beclin-1 has been proved to increase in patients with NB and is associated with other adverse prognostic factors (36). The expression of Beclin-1 activates autophagy and induces the malignant phenotype, promoting the expression of multidrug-resistant protein 2 and leading to chemoresistance (37), which is a poor prognostic factor of other tumors and negatively correlated with patient’s survival (36, 38, 39).

This study provided a theoretical basis for overcoming chemoresistance in patients with NB. Increasing the intensity of chemotherapy within a safe range can reduce the incidence of chemoresistance. It is possible to avoid the occurrence of autophagy-related chemoresistance by substituting cyclophosphamide with ifosfamide with a similar antitumor effect when developing a multi-drug combination regimen for NB. Replacement of cisplatin and VP16 in the same way can potentially benefit the patients. Conventional chemotherapy combined with CQ or Beclin-1 targeted agents may be used as a new regimen for NB; however, further prospective clinical studies are warranted to confirm this finding.

This study has some limitations. The clinical study is retrospective in nature, and the research objects are the patients; hence, the tumor tissues cannot be obtained for molecular biological study at each treatment time point to confirm the occurrence of chemoresistance. The chemoresistance rate can only be roughly estimated through a clinical efficacy evaluation. Ifosfamide and cyclophosphamide have similar antitumor mechanisms, but the effect of inducing NB autophagy is different, which cannot be explained for the time being and needs further study.

In conclusion, the chemoresistance rate of high-risk NB gradually increases during induction therapy, which has a potential impact on patients’ subsequent treatment and outcomes. Increasing the intensity of chemotherapy or inhibiting the occurrence of autophagy in tumor cells can partly overcome chemoresistance.

## Data availability statement

The datasets presented in this study can be found in online repositories. The name of the repository and the accession number can be found below: Research Data Deposit at https://www.researchdata.org.cn, reference number RDDA2022855006 (accessed on 13 May 2022).

## Ethics statement

The study involving human participants was approved by the ethics Committee of Sun Yat-sen University of Cancer Center (approval no. B2022-291-01), and the study was conducted according to the principles of the Declaration of Helsinki. All children’s guardians signed an informed consent.

## Author contributions

Conceptualization: TC, ZZ. Methodology: TC, CZ, ZZ. Software: TC. Validation: TC, CZ and ZZ. Formal analysis: TC and CZ. Investigation: JW, FS, JH, SL, JZ and YZ. Data curation: TC and CZ. Writing—original draft preparation: TC. Writing—review and editing: TC, ZZ. Supervision: ZZ, XS and YZ. Project administration: ZZ. All authors have read and agreed to the published version of the manuscript. All authors contributed to the article and approved the submitted version.

## Funding

This work was supported by a grant from the Science and Technology Planning Project of Guangdong Province, China (2018A030313838). The funders had no role in study design, data collection and analysis, decision to publish, or preparation of the manuscript.

## Conflict of interest

The authors declare that the research was conducted in the absence of any commercial or financial relationships that could be construed as a potential conflict of interest.

## Publisher’s note

All claims expressed in this article are solely those of the authors and do not necessarily represent those of their affiliated organizations, or those of the publisher, the editors and the reviewers. Any product that may be evaluated in this article, or claim that may be made by its manufacturer, is not guaranteed or endorsed by the publisher.
